# The relationship between the use of artificial sweeteners and cancer: A meta‐analysis of case–control studies

**DOI:** 10.1002/fsn3.2395

**Published:** 2021-06-23

**Authors:** Liping Liu, Peng Zhang, Yuanxin Wang, Weiwei Cui, Dong Li

**Affiliations:** ^1^ Department of Immunology College of Basic Medical Sciences Jilin University Changchun China; ^2^ Department of Thoracic Surgery The First Hospital of Jilin University Changchun China; ^3^ Department of Nutrition and Food Hygiene School of Public Health Jilin University Changchun China

**Keywords:** artificial sweetener, cancer, case–control studies, meta‐analysis

## Abstract

Although there are reports that artificial sweeteners (AS) are safe, the relationship between artificial sweeteners and cancer remains controversial. The purpose of the study is to evaluate whether the consumption of artificial sweeteners is associated with the risk of cancers. We conducted a comprehensive search of multiple databases, including MEDLINE, EMBASE, Web of Science, and Cochrane Library. We found all the literature that studied the relationship between artificial sweeteners and cancer. Ten case–control studies were included in the meta‐analysis. Our findings indicated that the consumption of artificial sweeteners was not associated with an increase in cancer when all types of cancers are analyzed comprehensively (OR 0.91, 95% CI 0.75–1.11). Interestingly, the use of artificial sweeteners is inversely related to urinary system cancer risk when analyzing women individually (OR 0.76, 95% CI 0.60–0.97). Our meta‐analysis found that these is no correlation between artificial sweeteners and occurrence of cancer except urinary system cancer in women. Considering some limitations found in this study, additional data from large clinical trials are needed.

## Graphical abstract

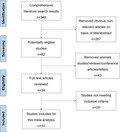

## 1 INTRODUCTION

Artificial sweetener refers to a compound that can be used as an additive in food and beverages to replace sugar (Weihrauch & Diehl, 2004). Saccharin, aspartame, cyclamate, and acesulfame potassium are currently popular artificial sweeteners (Kamenickova et al., 2013). As consumers pay more attention to how to reduce energy intake, artificial sweeteners are becoming more and more popular (Sakurai et al., 2014), and their use in food is also increasing, partly because they contain no calories, which can be used to control weight and obesity (Qurrat‐ul and Khan, 2015; Wiebe et al., 2011). A key question is whether replacement of sugar‐sweetened products with those containing artificial sweeteners has harmful effects at all. Although artificial sweetener is widely used throughout the world, people have been worried about its possible carcinogenic effects for a number of years (Weihrauch & Diehl, 2004). Recently, the morbidity and mortality of cancer in developing countries have risen (Pourhoseingholi et al., 2017), and the diagnosis and treatment of cancer have imposed a huge burden on the families and the health system (Matsuda & Saika, 2012).

The results of a study indicated that the heavy use of artificial sweeteners will increase the relative risk of bladder cancer in humans (Weihrauch & Diehl, 2004). Olivier et al. (2015) also suggested that nonsugar sweetener use could increase the risk of cancer. However, a human‐based study showed that a significant inverse trend in risk for increasing categories of total sweeteners was found for breast and ovarian cancer and a direct one for laryngeal cancer (Gallus et al., 2007). In addition to human research, there is also research on animals. The results of a meta‐analysis on the carcinogenic effects of aspartame on rodents showed that the consumption of aspartame will not have a significant carcinogenic effect on rodents (Mallikarjun & Sieburth, 2015). Although it has been announced that artificial sweeteners used in foods can be safely used as long as they are below their acceptable daily intakes, respectively, the carcinogenic effects of artificial sweeteners still remain controversial. This meta‐analysis summarized data on artificial sweeteners and various cancers to determine the relationship between artificial sweeteners and cancer.

## 2 MATERIALS AND METHODS

### 2.1 Sources and methods of data retrieval

We searched the PubMed, Cochrane library, Web of Science, and EMBASE databases from the inception dates to April 2021. The following terms were used to identify published literature evaluating the effect of artificial sweeteners on cancer: artificial sweetener, non‐nutritive sweeteners, aspartame, saccharin, cyclamate, stevia, sucralose, acesulfame, cancer, and tumor. The term “OR” was used as the set operator to combine different sets of results. The literature search was limited to English language and human subjects.

### 2.2 Inclusion criteria and exclusion criteria

**FIGURE 1:**
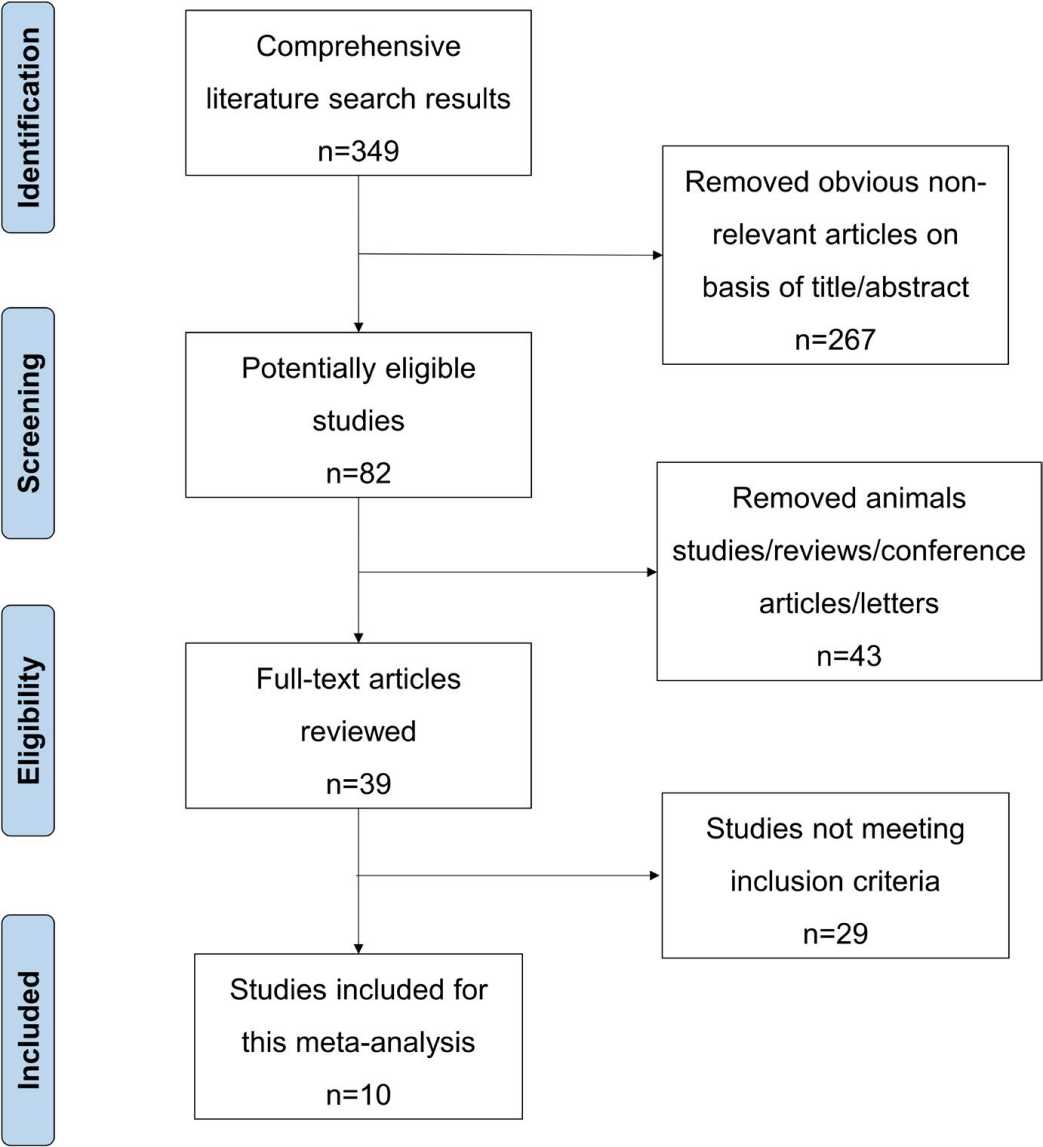
Flowchart showing the process for selection of eligible studies

The included articles need to meet the following six inclusion conditions: (1) Patients were clinically diagnosed; (2) article was published in peer reviewed journals in the English language; (3) the article contains initial data on artificial sweetener consumption and cancer risk; (4) the article needs to report the number of people using artificial sweeteners; (5) the outcomes were quantitative data that could be extracted or calculated; and (6) only include case–control studies. The following three exclusion criteria were applied: (1) We excluded studies that did not provide initial data, animal studies, in vitro studies, reviews, letters, personal opinions, book chapters, and conference abstracts; (2) only show the consumption of drinks containing artificial sweeteners; and (3) studies that full paper copy were not available. Two investigators independently reviewed the literature, extracted all potentially eligible studies, and resolved uncertainty and disagreement by discussion (Figure 1).

### 2.3 Data extraction

We reviewed all of the relevant studies and extracted the following data: (1) first author, nationality, publication year, numbers, and age of case subjects and the control group; and (2) artificial sweetener type, the number of people exposed to artificial sweeteners in case and control groups, and the type of cancer. Any differences related to the data extraction were resolved by rechecking the full text of the study or by discussion. When study data were ambiguous or data were not reported in a form that could be used for formal comparison, we contacted the corresponding and first author of the original publication via email.

### 2.4 Risk of bias within individual studies

The methodological quality for the selected literature was evaluated independently by two investigators according to the Newcastle–Ottawa scale (NOS) (Stang, 2010). The NOS contains eight items, categorized into three dimensions including selection, comparability, and outcome (cohort studies) or exposure (case–control studies). Each quality item has one star, and a study can get nine stars at most. The investigators resolved inconsistencies by discussion and consensus.

### 2.5 Statistical analysis

Statistical analysis was performed using the statistical software Stata (version 12.0, StataCorp LLC). Normalization test showed that the data included in this article do not conform to the normal distribution (*p* < .05). We calculated the odds ratios (ORs) and their respective 95% confidence intervals for case–control studies. The random‐effect model was used to compute OR and 95% confidence intervals (CIs) and to assess the differences of artificial sweetener exposure between the case group and control group. Cochran's Q statistic and the *I* ^2^ statistic were used to assess the statistical heterogeneity in the meta‐analysis (Cochran, 1954). If the data were homogeneous (*p* > .05), a fixed‐effect model meta‐analysis was performed; if the data were heterogeneous (*p* ≤ .05), a random‐effects model meta‐analysis was performed. In the Q test, *p* < .05 was considered significant for heterogeneity, and the *I* ^2^ value was used to evaluate the degree of heterogeneity. *I* ^2^ values of 25%, 50%, and 75% indicate low, moderate, and high heterogeneity, respectively (Higgins et al., 2003). The potential publication bias was evaluated via the Egger test, where the sensitivity analysis was used to correct outcomes and evaluate the impact of bias on the outcomes. Subgroup analyses were conducted based on the cancer type, age, and gender of subjects. Because most of the region in the included literature are different, no subgroup analysis was conducted for the region.

## 3 RESULTS

**FIGURE 2:**
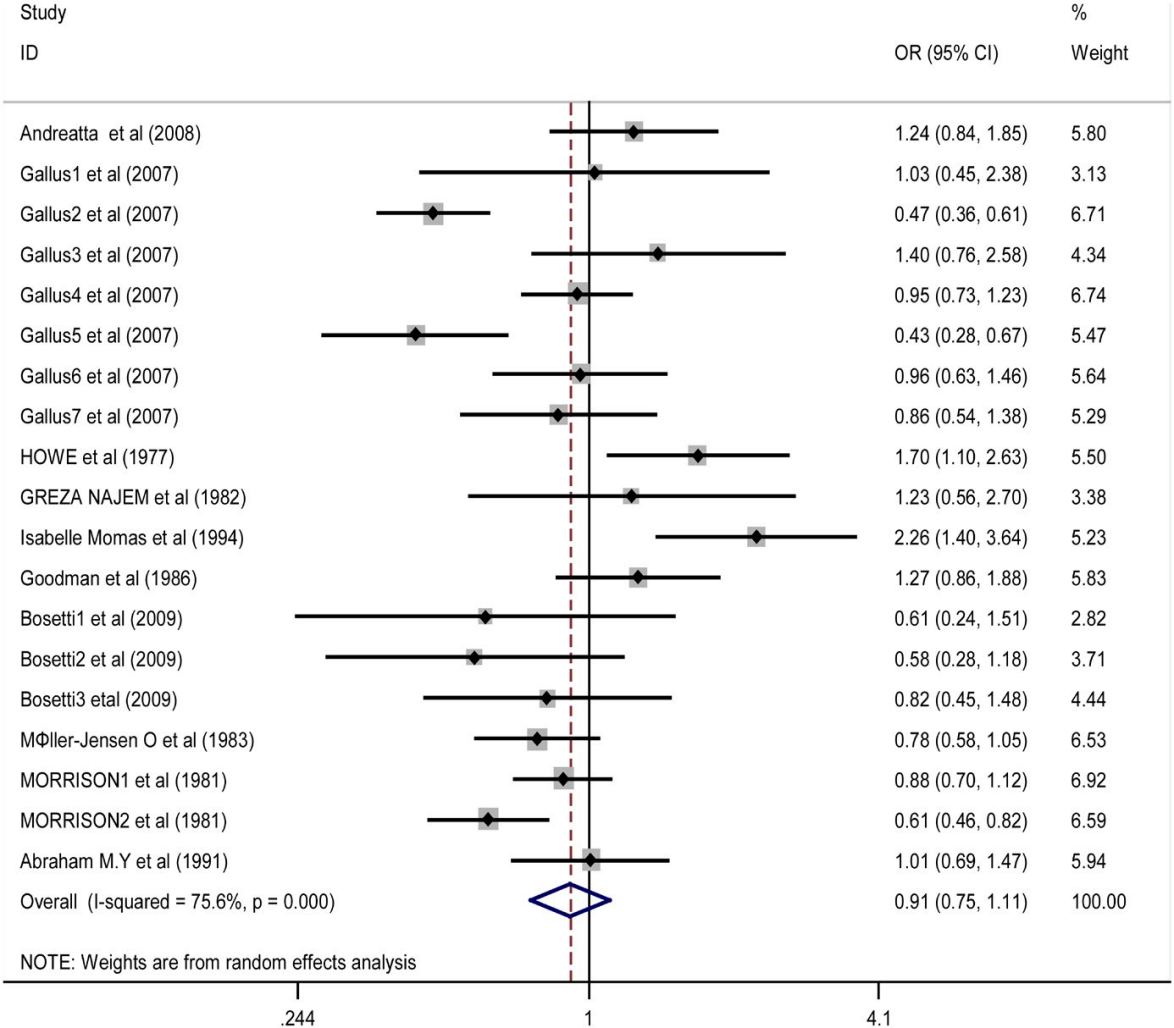
Forest plot describing the association of AS with risk for cancer (10 study arms). ORs of individual studies are indicated by the data markers; shaded boxes around data markers reflect the statistical weight of the study; 95% CIs are indicated by the error bars; and OR with their 95% CI is depicted as a diamond

Our study identified 349 related references, but only 10 papers met our inclusion criteria. These 10 articles included a total of 32,738 samples, with 12,052 cases and 20,686 controls (Andreatta et al., 2008; Bosetti et al., 2009; Gallus et al., 2007; Goodman et al., 1986; Howe et al., 1977; Møller‐Jensen et al., 1983; Momas et al., 1994; Morrison et al., 1982; Najem et al., 1982; Nomura et al., 1991). Most of the articles researched the effect of saccharine on cancer, and two of the articles researched the effect of artificial sweeteners on several cancer types (Bosetti et al., 2009; Gallus et al., 2007). Results were pooled by the type of cancer in all studies, and the detailed results are shown in Table 1. On quality assessment, the included studies had an NOS (Table S2) score of 6–7. Egger test (*t* 1.15, *p* > .05) showed that the effect of publication bias was considered slight (Figure. S1). The results of sensitivity analysis showed that there is no significant effect on the combined odds ratio (OR) value after excluding a certain study. The 10 case–control studies calculated ORs, comparing the risk of cancer between nonconsumers of artificial sweeteners and users of artificial sweetener. “Any” compared with “no” consumption of artificial sweeteners was not associated with cancer (OR 0.91, 95% CI 0.75–1.11). To analyze the source of heterogeneity, subgroup analysis was performed according to the type of cancer, the age, and gender of subjects (Figure 2).

**TABLE 1:** Basic characteristics of the studies included in the meta‐analysis

Author	Region	Year	Sweetener	Age		*N*		Gender (male/female)		*n*		Gender (male/female)		Cancer
				Case	Control	Case	Control	Case	Control	Case	Control	Case	Control	
Andreatta et al. (2008)	Argentina	2008	All	–	–	197	397	156/41	267/130	51	87	39/12	37/50	Urinary tract cancer
Gallus et al. (2007)	Italy	2006	Saccharine	60	60	304	743	275/29	593/150	8	19	–	–	Esophagus
Gallus et al. (2007)	Italy	2006	Saccharine	62	58	1,953	4,154	1125/828	2073/2081	69	300	–	–	Colorectum
Gallus et al. (2007)	Italy	2006	Saccharine	61	61	460	1,088	415/45	863/225	17	29	–	–	Larynx
Gallus et al. (2007)	Italy	2006	Saccharine	55	56	2,569	2,588	0/2569	0/2588	113	120	–	–	Breast
Gallus et al. (2007)	Italy	2006	Saccharine	56	57	1,031	2,411	0/1031	0/2411	24	126	–	–	Ovary
Gallus et al. (2007)	Italy	2006	Saccharine	66	63	1,294	1,451	1294/0	1451/0	42	49	–	–	Prostate
Gallus et al. (2007)	Italy	2006	Saccharine	62	62	767	1,534	494/273	988/546	26	60	–	–	Renal cell carcinoma
Howe et al. (1977)	Canada	1977	Saccharine	67.8	68.9	455	455	455/0	455/0	58	36	–	–	Bladder cancer
Najem et al. (1982)	US	1982	Saccharine	–	–	75	142	65/10	130/20	12	19	–	–	Bladder cancer
Momas et al. (1994)	France Mediterranean	1994	Saccharine	67.8	65.0	219	792	219/0	792/0	30	52	–	–	Bladder cancer
Goodman et al. (1986)	US	1986	saccharine	20–80	20–80	266	266	189/77	189/77	73	61	47/26	41/20	Renal cell cancer
Bosetti et al. (2009)	Italy	2009	Saccharine	63	63	230	544	–	–	6	23	–	–	Gastric cancer
Bosetti et al. (2009)	Italy	2009	Saccharine	63	63	326	652	–	–	10	34	–	–	Pancreatic cancer
Bosetti et al. (2009)	Italy	2009	Saccharine	60	61	452	906	–	–	16	39	–	–	Endometrial cancer
Møller‐Jensen et al. (1983)	Copenhagen	1983	Saccharine, cyclamate	0–75	0–75	380	776	284/96	583/193	81	200	55/26	150/50	Bladder cancer
Morrison et al. (1982)	UK	1981	Saccharine	–	–	524	690	382/142	470/220	190	270	140/50	183/87	Bladder cancer
Morrison et al. (1982)	Japan	1981	Saccharine	–	–	289	576	223/66	432/144	126	321	100/26	238/83	Bladder cancer
Ahmad et al. (2001)	Japan	1991	Saccharine	68.2	68.2	261	521	195/66	389/132	50	99	40/10	72/27	Urinary tract cancer

**FIGURE 3:**
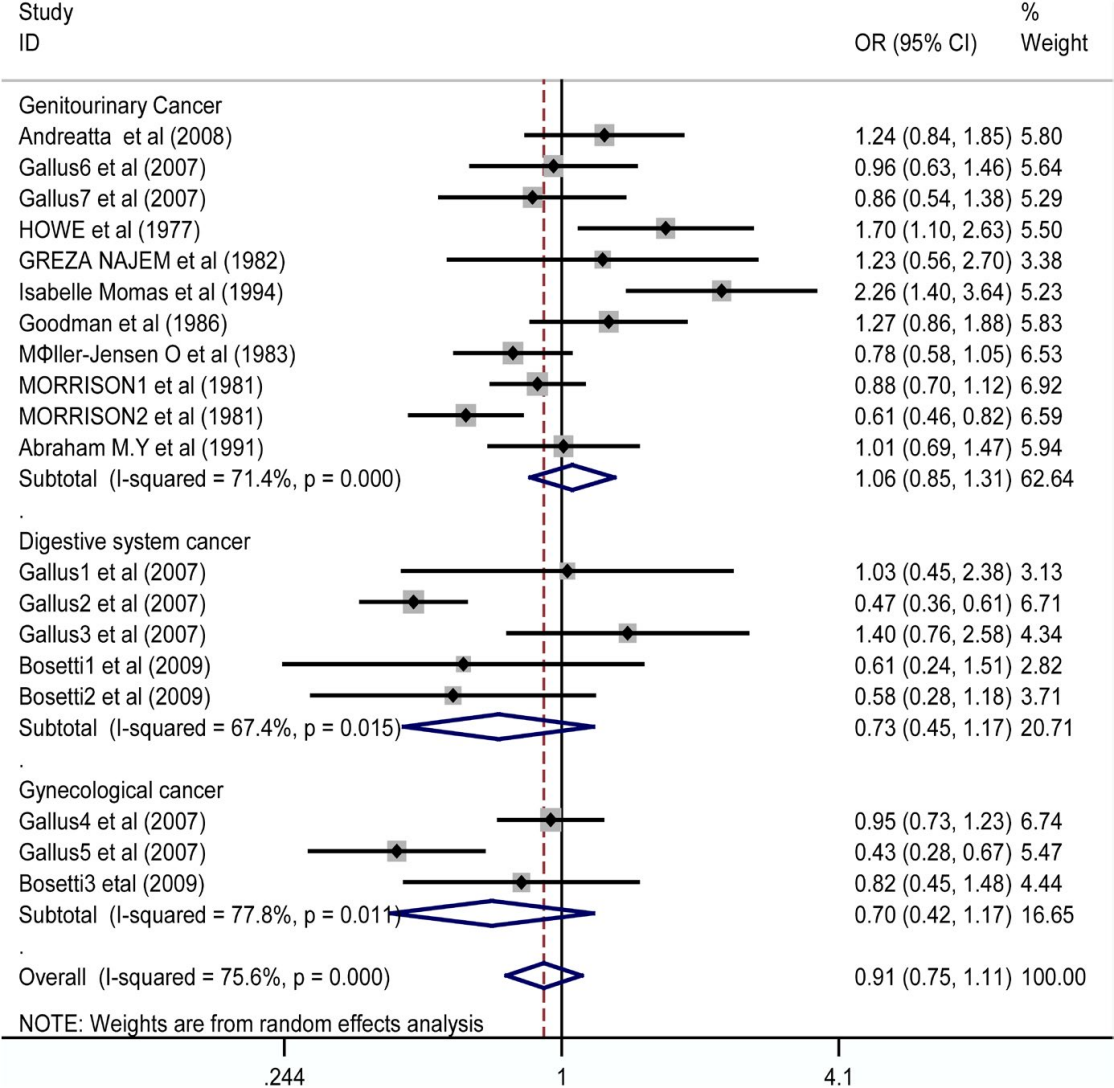
Forest plot for the subgroup of cancer. ORs of individual studies are indicated by the data markers; shaded boxes around data markers reflect the statistical weight of the study; 95% CIs are indicated by the error bars; and OR with their 95% CI is depicted as a diamond

Subgroup analysis of cancer: The results of subgroup analysis of cancer indicated that the risk of digestive system cancer (OR 0.73, CI 0.45–1.17) (Bosetti et al., 2009; Gallus et al., 2007), genitourinary system cancer (OR 1.06, CI 0.85–1.31) (Andreatta et al., 2008; Gallus et al., 2007; Goodman et al., 1986; Howe et al., 1977; Møller‐Jensen et al., 1983; Momas et al., 1994; Morrison et al., 1982; Najem et al., 1982; Nomura et al., 1991), and gynecological cancer (OR 0.70, CI 0.42–1.17) (Bosetti et al., 2009; Gallus et al., 2007) are not related to the use of artificial sweeteners (Figure 3).

**FIGURE 4:**
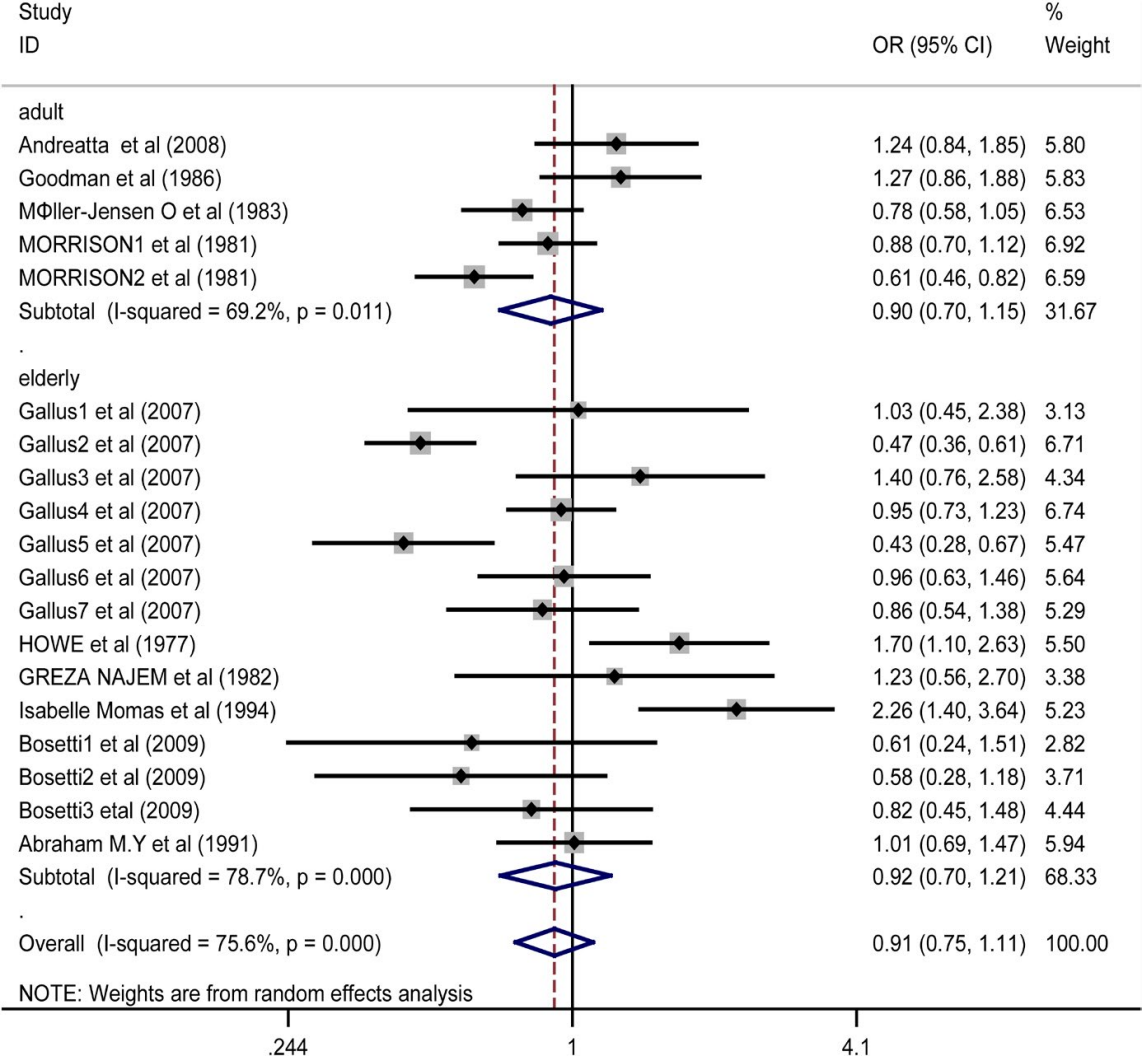
Forest plot for the subgroup of age. ORs of individual studies are indicated by the data markers; shaded boxes around data markers reflect the statistical weight of the study; 95% CIs are indicated by the error bars; and OR with their 95% CI is depicted as a diamond

Subgroup analysis of age: We divided the studies into two subgroups according to the age of subjects, and the subgroup analysis showed that the risk of cancer was not related to the use of artificial sweeteners in adult (adult's age range is 18–60 years (Ahmad et al., 2001), OR 0.90, CI 0.70–1.15) (Andreatta et al., 2008; Goodman et al., 1986; Møller‐Jensen et al., 1983; Morrison et al., 1982) and elderly (elderly is above 60 years old (Ahmad et al., 2001), OR 0.92, CI 0.70–1.21) (Bosetti et al.,^2009^; Gallus et al., 2007; Howe et al., 1977; Momas et al., 1994; Najem et al., 1982; Nomura et al., 1991) (Figure 4).

**FIGURE 5:**
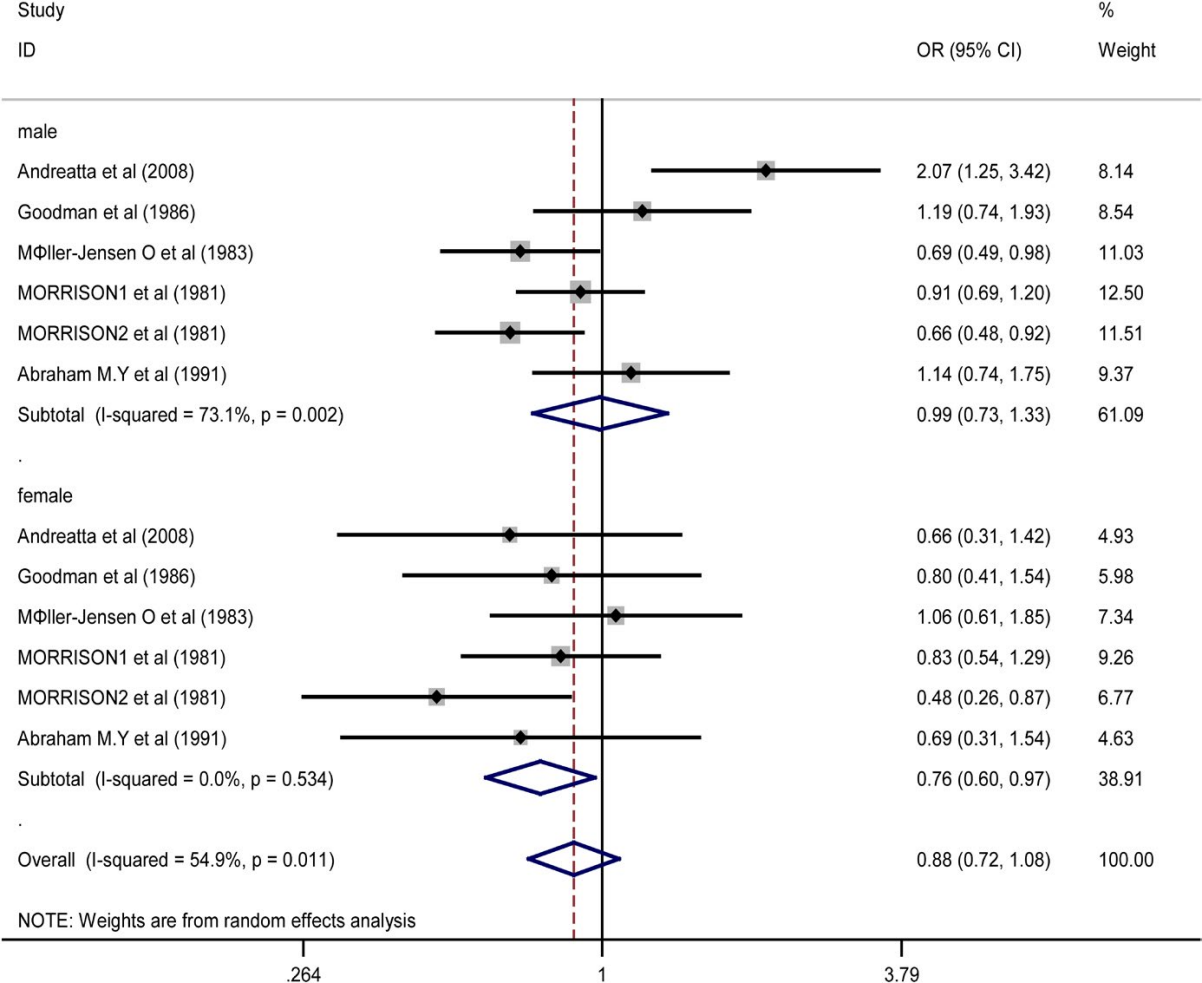
Forest plot for the subgroup of gender. ORs of individual studies are indicated by the data markers; shaded boxes around data markers reflect the statistical weight of the study; 95% CIs are indicated by the error bars; OR with their 95% CI is depicted as a diamond

Subgroup analysis of gender: In the included study, five articles investigated the number of men and women using artificial sweeteners (Ahmad et al., 2001; Goodman et al., 1986; Møller‐Jensen et al., 1983; Morrison et al., 1982; Nomura et al., 1991). When analyzing men and women separately, *I* ^2^ decreased to 0% among women, and the statistical analysis results are shown in Figure 5. The use of artificial sweeteners is not related to the risk of cancer in men (OR 0.99, CI 0.73–1.33) (Andreatta et al., 2008; Goodman et al., 1986; Møller‐Jensen et al., 1983; Morrison et al., 1982; Nomura et al., 1991); however, the use of artificial sweeteners is inversely related to the risk of urinary system cancer in women (OR 0.76, 95% CI 0.60–0.97) (Andreatta et al., 2008; Goodman et al., 1986; Møller‐Jensen et al., 1983; Morrison et al., 1982; Nomura et al., 1991).

## 4 DISCUSSION

We included 10 case–control studies, which assessed the association between artificial sweeteners and different types of cancer. For most outcomes, there seemed to have no statistical difference between artificial sweeteners intake and nonintake. But it can be seen that the use of artificial sweeteners is inversely related to the risk of urinary system cancer in women.

This meta‐analysis included studies of multiple types of cancer and focused on discussing the relationship between artificial sweeteners and cancer. By analyzing the forest plots, we can find that when all types of cancer were analyzed together, the difference in result was not statistically significant. From the results of the subgroup analysis, it can be seen that the results were not statistically significant in the subgroup analysis of cancer type and age. However, when males and females are analyzed separately, heterogeneity drops from 54.9% to 0%, which indicating that gender may be one of the reasons for the heterogeneity. Additionally, the geographical differences of the research subjects (the artificial sweetener consumption in Italy is very low (Leclercq et al., 1999), and the artificial sweetener exposure rate in Argentina is high (Andreatta et al., 2008) will also affect the results. Due to cancer is an age‐related disease (Kendal, 2008), the age of subjects may be also an important confounding variable.

From subgroup analyses of gender, we saw that the results were different in men and women. The use of artificial sweeteners is not related to the risk of cancer in men (OR 0.99, CI 0.73–1.33); however, the use of artificial sweeteners is inversely related to the risk of urinary system cancer in women (OR 0.76, 95% CI 0.60–0.97), which is different from previous research. The reason for this result may be that the sample size of women is small or that the number of women in the case group is less than one‐third of the number of men or the number of women in the control group is less than half of the number of women. In addition, the short average use time of artificial sweeteners in the population included in the study may also be a reason. From the research data of urinary system cancer, it can be found that among the population included in the study, there are fewer women than men, which reflects from the side that the lower prevalence of female urinary system cancer. A researcher Morrison et al. (1982) found that random variability or unrecognized deviations may be the reason for the inverse relationship between artificial sweeteners and bladder cancer in Nagoya. It is still uncertain whether the use of artificial sweeteners can reduce the risk of urinary system cancer in women, so more research is needed to verify this result.

In addition to observational research, there are people who have conducted genetic research. One study reported the interaction of aspartame and its metabolites with DNA in an in vitro model (Karikas et al., 1998) and another study showed that aspartame may induce DNA strand breaks in mouse bone marrow cells Bandyopadhyay et al. (2008), so it can be considered that aspartame is potentially carcinogenic. As early as 1970, a study found that sweetened sodium and saccharin sodium increased the incidence of bladder tumors in rats, indicating that the use of artificial sweeteners may increase the risk of cancer (Wagner, 1970), and it was later suggested that cyclamate has adverse effects on the testes of rats (Renwick, 1986). After that, more and more researchers began to pay attention to the effects of artificial sweeteners. The relationship between artificial sweeteners and cancer is a difficult subject to study because the types of sweeteners and cancer are very wide. Compared with population research, animal research is easier to conduct, and many scientists have studied the effects of artificial sweeteners on animals. In 1996, researchers proposed a link between artificial sweeteners and cancer in mice, which indicated that aspartame was associated with brain tumor (Olney et al., 1996). Subsequent experiments on monkeys showed that the use of saccharin was not associated with an increased risk of cancer (Takayama et al., 2000), but when trying to extrapolate animal data to humans, care must be taken because the carcinogenic mechanisms differ between humans and animals (International Agency for Research on Cancer, 1999).

The relationship between the consumption of artificial sweeteners and cancer is a complex research topic, because the range of artificial sweeteners and cancer is very wide. Human data on artificial sweetener intake and cancer risk are scarce and largely have not been supportive of an association between artificial sweetener intake and cancer risk (Bosetti et al., 2009; Lim et al., 2006; McCullough et al., 2014; Mishra et al., 2015). Although data from long‐term human studies are lacking, a large amount of short‐term and animal evidence seems to prove that artificial sweetener has no health effects. Lim et al. (2006) showed that the consumption of aspartame‐containing beverages was not related to the incidence of hematopoietic and brain malignancies; moreover, research by McCullough et al. (2014) showed that consumption of artificial sweeteners is not related to the risk of lymphoma in the elderly. Most recently, data presented in a systematic review do not conclusively support the carcinogenicity of artificial sweeteners (Mishra et al., 2015).

Most of the previous research was on the relationship between artificial sweeteners and urinary system cancer. Toews et al. (2019) analyzed the relationship between nonsugar sweeteners and cancer when studying the health effects of nonsugar sweetener, and the results showed that the risk of bladder or lower urinary tract cancer seemed to be similar between those exposed to sweeteners and those unexposed to sweeteners. Møller‐Jensen et al. (1983) indicated that the consumption of artificial sweeteners is unlikely to be associated with any appreciable increase in bladder cancer risk. Nomura et al. (1991) demonstrated that there was no indication that the use of saccharin or artificial sweeteners in diet beverages was strongly related to bladder cancer risk. Although Morrison et al. (1982) observed an inverse relationship between artificial sweeteners and bladder cancer in Nagoya, this may be the result of random variability or unrecognized bias. In addition, Goodman et al. (1986) showed that no significant differences between cases and controls were found for either the amount or duration of artificial sweetener use or the lifetime consumption of saccharin. However, Andreatta et al. (2008) found that the use of AS was positively associated with urinary tract tumors risk only when consumed regularly for 10 years or more, which is consistent with previous research (Sturgeon et al., 1994). Nevertheless, the mechanisms behind the artificial sweetener‐related urinary system cancer in women are largely unknown or whether this phenomenon is debatable. More large‐scale studies and investigations into the underlining mechanisms are required for us to understand this issue.

Case–control studies have some inherent shortcomings, such as more research bias and confounding effects. Due to the small amount of literature on the relationship between artificial sweeteners and cancer, this meta‐analysis has certain limitations. Only 10 case–control studies with initial data were retrieved, the heterogeneity of result is high, and the reason may be the differences of subjects. In addition, this article used “yes” or “no” to determine whether artificial sweeteners have been consumed, without considering the consumption of amount and time of artificial sweeteners. Importantly, the scores of the INOS scale are not high. A previous researcher suggested that future research should evaluate the health effects of using artificial sweeteners with appropriate research time (Toews et al., 2019). The results of observational studies on the health effects of nonsugar sweeteners should be interpreted with caution, and attention should be focused on possible residual confounding and reverse causality (Sievenpiper et al., 2017). Whether artificial sweeteners increase the risk of cancer and whether it has a protective effect on urinary system cancer, which requires more and longer‐term research to determine.

## 5 CONCLUSION

In summary, there is no sufficient evidence to show whether the use of artificial sweeteners increases or decreases the risk of cancer. Considering some limitations found in this study, more data from large clinical trials are needed to affirm the relationship between artificial sweeteners and cancer.

## CONFLICTS OF INTEREST

The authors declare that there are no conflicts of interest regarding the publication of this paper.

## AUTHOR CONTRIBUTIONS

WC, DL, LL, and PZ made the study design. LL and PZ conducted the study. LL, PZ, and YW analyzed the data and wrote the manuscript. LL, PZ, and YW attended the manuscript revision. All authors agreed with the final manuscript.

## ETHICAL APPROVAL

Ethical Review: This study does not involve any human or animal testing. Ethics approval was not required for this research.

## Supporting information

Supplementary Material

## Data Availability

We confirm that the data supporting the findings of this study are available within the article.
