# Supplementary material for: The relationship between the use of artificial sweeteners and cancer: A meta‐analysis of case–control studies
Source: Food Sci Nutr. 2021 Jun 23;9(8):4589–97. doi: 10.1002/fsn3.2395 (PMC8358351; doi:10.1002/fsn3.2395)
Supplement: Supplementary file 1 — Supplementary Material [file FSN3-9-4589-s001.docx]

Table 2. Quality assessment of the papers included in the systematic review.

| Author | year | Selection  (max 4) | Comparability  (max 2) | Exposure  (max 4) | Total  (max 10) |
| --- | --- | --- | --- | --- | --- |
| Andreatta et al^17^ | 2008 | 3 | 2 | 1 | 6 |
| Gallus et al^9^ | 2006 | 3 | 2 | 1 | 6 |
| HOWE et al^21^ | 1977 | 4 | 1 | 1 | 6 |
| GREZA NAJEM et al^19^ | 1982 | 3 | 2 | 1 | 6 |
| Momas et al^22^ | 1994 | 4 | 2 | 1 | 7 |
| GoodMan et al^20^ | 1986 | 3 | 2 | 1 | 6 |
| Bosetti et al^18^ | 2009 | 3 | 2 | 1 | 6 |
| MØLLER-JENSEN et al^14^ | 1983 | 4 | 2 | 1 | 7 |
| MORRISON et al^15^ | 1981 | 4 | 2 | 1 | 7 |
| Abraham M.Y et al^16^ | 1991 | 4 | 2 | 1 | 7 |


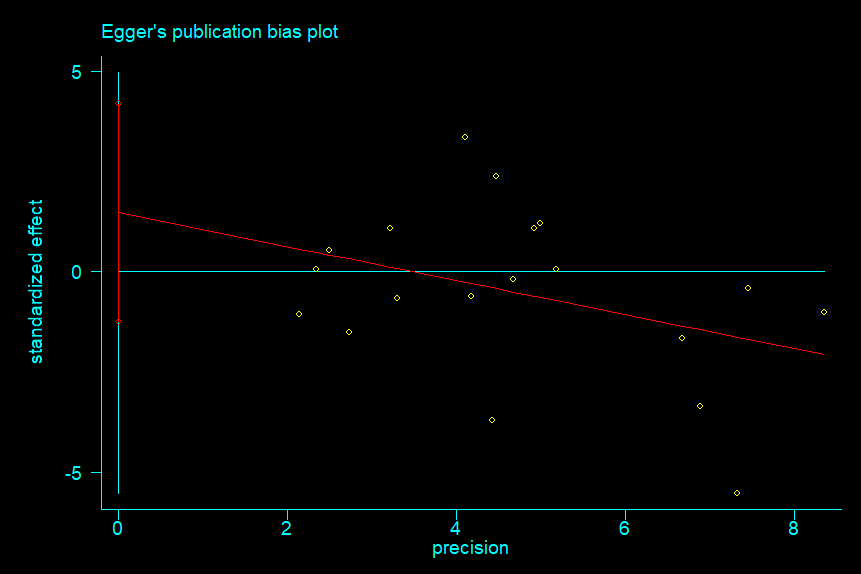


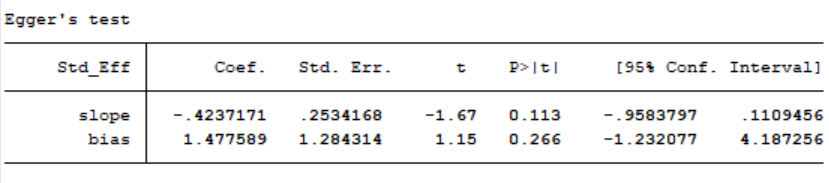


Figure.s1 Egger’s publication bias plot and its result. The Coef in the Egger test is 1.477589 and it is the intercept of the regression analysis, and -1.232077 and 4.187256 are the 95% confidence intervals of this intercept. The slope is -0.4237171 and its 95% confidence interval is -0.9583797nand 0.1109456. The index of P>|t| in the row of Bias is 0.266, which means that there is no obvious bias in this study.
